# A Reproductive Management Program for an Urban Population of Eastern Grey Kangaroos (*Macropus giganteus*)

**DOI:** 10.3390/ani4030562

**Published:** 2014-09-15

**Authors:** Andrew Tribe, Jon Hanger, Ian J. McDonald, Jo Loader, Ben J. Nottidge, Jeff J. McKee, Clive J. C. Phillips

**Affiliations:** 1 School of Agriculture and Food Sciences, University of Queensland, Gatton, Queensland 4343, Australia; E-Mails: a.tribe@uq.edu.au (A.T.); ianmcd85@hotmail.com (I.J.M.); 2 Endeavour Veterinary Ecology Pty Ltd, Toorbul, Queensland 4510, Australia; E-Mails: jonhanger@hotmail.com (J.H.); jo@endeavourvet.com.au (J.L.); 3 GreenLeaf Ecology, Mooloolah, Queensland 4553, Australia; E-Mail: ben@glecology.com.au; 4 Ecosure, West Burleigh, Queensland 4219, Australia; E-Mail: jmckee@ecosure.com.au; 5 Centre for Animal Welfare and Ethics, University of Queensland, Gatton, Queensland 4343, Australia

**Keywords:** behaviour, Deslorelin, orchidectomy, vasectomy, welfare

## Abstract

**Simple Summary:**

We designed a programme to control free-ranging kangaroos on a Queensland golf course, using contraceptive implants in females and vasectomisation or testicle removal in males. This reduced the numbers of pouch young to about one half of pre-intervention levels and controlled the population over a 2–4 year period. However, the necessary darting caused a mortality rate of 5–10% of captured animals, mainly due to complications before and after anaesthesia. It is concluded that population control is possible but careful management of kangaroos around the time of anaesthesia induction and recovery is important in such programmes to minimise losses.

**Abstract:**

Traditionally, culling has been the expedient, most common, and in many cases, the only tool used to control free-ranging kangaroo populations. We applied a reproductive control program to a population of eastern grey kangaroos confined to a golf course in South East Queensland. The program aimed to reduce fecundity sufficiently for the population to decrease over time so that overgrazing of the fairways and the frequency of human–animal conflict situations were minimised. In 2003, 92% of the female kangaroos above 5 kg bodyweight were implanted with the GnRH agonist deslorelin after darting with a dissociative anaesthetic. In 2007, 86% of the females above 5 kg were implanted with deslorelin and also 87% of the males above 5 kg were sterilised by either orchidectomy or vasectomy. In 2005, 2008 and 2009, the population was censused to assess the effect of each treatment. The 2003 deslorelin program resulted in effective zero population growth for approximately 2.5 years. The combined deslorelin–surgery program in 2007 reduced the birth rate from 0.3 to 0.06%/year for 16 months, resulting in a 27% population reduction by November 2009. The results were consistent with implants conferring contraception to 100% of implanted females for at least 12 months. The iatrogenic mortality rates for each program were 10.5% and 4.9%, respectively, with 50% of all mortalities due to darting-related injuries, exertional myopathy/hyperthermia or recovery misadventure. The short term sexual and agonistic behaviour of the males was assessed for the 2007 program: no significant changes were seen in adult males given the vasectomy procedure, while sexual behaviours’ were decreased in adult males given the orchidectomy procedure. It is concluded that female reproduction was effectively controlled by implantation with deslorrelin and male reproductive behaviour was reduced by orchidectomy, which together achieved population control.

## 1. Introduction

Loss and fragmentation of wildlife habitats and the ecological consequences that follow are major challenges for nature conservation. While loss of biodiversity is one of the most concerning trends, local overabundance, particularly in isolated habitat remnants, may lead to human–animal conflicts and undesirable ecological effects requiring intervention. It is important that management of overabundant species is effective, ecologically sound, humane and socially acceptable. 

Since European settlement, the eastern grey kangaroo (*Macropus giganteus*) is one Australian species that is said to have benefited from habitat changes, increasing in population size and range along the eastern coast and for some distance inland [1,2]. Within Australian society, the kangaroo is perceived both as an iconic national symbol and but also invasive in many areas, hence opinion regarding management options varies from full protection to unrestricted culling [3]. However, because of perceived animal welfare and ethical issues and increased availability of non-lethal options, culling is now under greater public scrutiny and socio-political restraint. Non-lethal management options, including translocation and partial or complete reproductive suppression of a population, have greater appeal and hence are the subjects of ongoing investigation [4,5,6,7,8]. 

Although non-lethal management options may be more socio-politically acceptable, there are nevertheless potential risks to animal welfare associated with capture, anaesthesia and surgical procedures that must be mitigated if such methods are to be widely accepted. Furthermore, behavioural changes associated with pharmacological or surgical intervention may affect the dominance hierarchy and social structure within a population [9,10,11,12]. This may reduce group cohesion, which increases their vulnerability in a semi-porous containment area, such as a golf course. Reduced sexual activity may in particular reduce group cohesion. 

Herbert *et al.* [5] demonstrated that the gonadotrophin-releasing hormone (GnRH) agonist deslorelin is an effective contraceptive in female eastern grey kangaroos. In their study, the mean contraceptive period for sustained-release implants containing 10 mg of deslorelin was approximately 18 months. Nave *et al.* [8] and Couslon *et al.* [6] demonstrated periods of contraception of approximately 27–48 months in female eastern grey kangaroos after treatment with levonorgestrel implants. 

Surgical sterilisation results in permanent reproductive impairment, but is more invasive, labour intensive and requires specialist training. Male vasectomy and distal oviductal transection of females are two methods that have been used to control fecundity in the Kangaroo Island koala population successfully [13]. Castration has not, to our knowledge, been used for suppression of reproduction in wild kangaroo populations, but orchidectomy and vasectomy are routine, safe procedures used for sterilising male captive macropods in zoological collections [14] and to our knowledge have been used in one urban population of kangaroos in Canberra [15]. At present, the behavioural and social effects of a reproductive management program involving a variety of fertility control techniques, such as hormonal contraception and surgical sterilisation, in wild kangaroos have not been documented.

The aim of the reproductive management program reported here was to achieve zero or negative growth in a kangaroo population using a combination of fertility control methods, namely the use of deslorelin implants in females and surgical sterilisation of males using orchidectomy or vasectomy. The mean yearly percentage reduction in recruitment, the effective population growth rates and the cost per percent reduction in recruitment achieved using deslorelin alone are reported and compared with a combination of deslorelin and surgical sterilisation. In addition, the iatrogenic morbidity and mortality rates associated with capture and restraint and the percentage reduction in agonistic and sexual behaviours associated with vasectomy and orchidectomy have been summarised. 

## 2. Methods

### 2.1. Study Site

**Figure 1:**
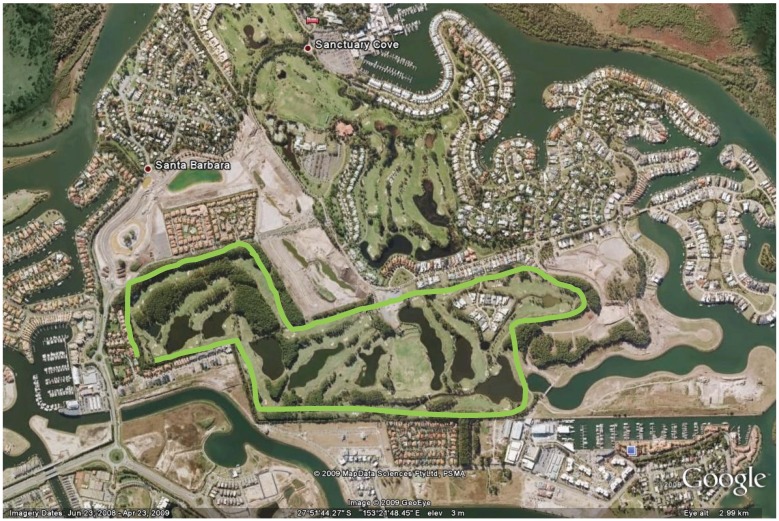
Map showing Pines golf course and surrounding urban areas (Google Earth, 2009).

This study was conducted on a 100 hectare golf course situated on the Gold Coast, Queensland, Australia (Figure 1). In 2003, the course was bounded on its northern and western sides by high density residential areas, on its eastern side by a marine inlet, and on its southern side by a grassland area under preparation for residential development. Since that time, the adjacent block on the southern side has been developed as a high-density residential precinct. The southern and western boundaries of the golf course were contained by a 2 m high chainmesh fence, porous in places. The course contained a number of artificial freshwater lakes and waterways and large and small “roughs” vegetated by stands of pine (*Pinus spp.*) or mixed native and exotic vegetation. 

In 2003, the managers of the golf course were concerned that the current kangaroo population size could increase the risk of human injury and cause damage to fairways and greens from overgrazing. Consequently, they elected to explore management options. Culling was not considered for ethical, public safety and public relations reasons.

### 2.2. The Eastern Grey Kangaroo Population

Notwithstanding the issues with fence integrity, the eastern grey kangaroo population was mainly confined to the golf course. The kangaroos followed a typical crepuscular activity pattern, and during the middle of the day rested in variably-sized mobs in some of the roughs. Anecdotally, the causes of premature mortality in the population were motor vehicle strike on adjacent roads and fox predation on young animals. The kangaroos were habituated to the presence of golfers and their buggies. For the purposes of this study, “flight distance” was defined as the minimum human to animal distance at which kangaroos took flight or displayed avoidance behaviour. We observed that for most kangaroos on the golf course flight distances were in the order of 5–10 m, although this distance increased to 10–35 m over the capture period. 

### 2.3. Census

An initial count of the kangaroo population was undertaken in 2003 by Wildcare Population Health Services. The estimated population at the time was believed to be 194 kangaroos. In 2007, two methods were used to count kangaroos and record basic demographic data:

Method 1: All kangaroos were observed with binoculars to determine sex, age group (adult, sub-adult) and presence of pouch young. This method was the slower of the two and increased the likelihood of double counting due to milling. 

Method 2: Kangaroos were counted rapidly irrespective of age and sex class and excluded pouch young. 

Kangaroo numbers were assessed from a moving buggy, which stopped if the numbers of kangaroos were too many to count instantaneously. Population estimates were based on the number counted by method 2 plus the number of pouch young observed by method 1, multiplied by a correction factor of 1.2 to allow for undetectable pouch young. This correction factor was based on our estimate that approximately one sixth of pouch young were too small to be detected during the census.

### 2.4. Capture Restraint and Reproductive Control

The first of two reproductive control programs occurred between August and September 2003. As many as possible of the adult and juvenile kangaroos more than 5 kg were captured by projectile anaesthesia, clinically assessed and tagged. Only females weighing more than 5 kg were implanted with deslorelin-containing slow-release implants (Suprelorin 12^®^, 9.4 mg deslorelin, Peptech Animal Health). Weight was established by experienced wildlife veterinarians after translocation of each animal. A follow-up census was conducted in November 2005. 

The second reproductive control program occurred between May and July 2007. All female kangaroos weighing more than 5 kg were implanted with Suprelorin and all adult and sub-adult male kangaroos more than 5 kg were surgically sterilised. Censuses were conducted in November 2008 and November 2009. A summary of the program timeframes is contained in Table 1. 

**Table 1:** Population management program timeframes.

Time	Activity
March 2003	Initial population estimate
August–September 2003	Capture and tag all kangaroos. Suprelorin implantation of females more than 5 kg only
November 2005	Population census
May–July 2007	Capture and tag all kangaroos. Surgical sterilisation of all males more than 5 kg. Suprelorin implantation of all females above 5 kg
March–September 2007	Behavioural observations
November 2008	Population census
November 2009	Population census

During the 2003 program, kangaroos demonstrated increasing flight distances as darting progressed. After 3 days of continuous capture the animals’ flight distances were beyond the safe range of the dart gun and the program was deferred for 2–3 weeks to allow the kangaroos to settle. Thereafter, each capture session occurred over only 2 days with breaks of 2–3 weeks between sessions. The 2003 program was completed in 5 capture days over a total elapsed time of 2 months. The 2007 program was completed in 6 capture days over a total elapsed time of 2 months.

### 2.5. Kangaroo Capture and Veterinary Procedures

#### 2.5.1. Capture and Anaesthesia

Kangaroos were captured by projectile anaesthesia using Tel-Inject or Dan-Inject re-useable darts fired from either a Taipan 2000 (Montech Industries) or JM Special 25 (Dan-Inject) dart rifle. The anaesthetic agent was Zoletil (tiletamine + zolazepam) (Virbac (Australia), NSW, Australia) delivered at 5–15 mg/kg body weight over darting distances of 5–35 m. Surgeries were performed using standard techniques in a temporary facility observing aseptic techniques. Additional anaesthetic was administered if required. Two darting teams were in operation, consisting of one veterinarian, one veterinary nurse, and one field biologist (spotter), along with kangaroo transport teams consisting of three groups of two wildlife carers which followed each darted kangaroo in a buggy until it was fully immobilised and therefore safe to move. Darting teams returned to the central management area to check on progress with each animal. A veterinary procedures team, consisting of one veterinarian, two veterinary nurses and three volunteer wildlife carers assisted with animal handling and general duties. On arrival, the kangaroo was weighed and placed on the surgery table for a full physical examination and prepared for surgery. A data sheet was then commenced and accompanied the animal to the veterinary procedures area in the golf cart, taking up to 5 minutes. 

In 2003, all darted kangaroos were tagged with swivel-type plastic numbered ear tags (Swing-free Tags, Stockbrands, WA, Australia) and marked with temporary stock marking paint to prevent accidental re-darting. Prior to surgery, each kangaroo was given a physical examination to check body condition and gross evidence of any injury or illness, and a cloacal temperature measurement was taken every 15 minutes. Each captured kangaroo was given an injection of Vitamin E + selenium (Ilium Selvite E, Troy Laboratories, Smithfield, NSW, Australia) at a dose rate of 1 mL/30 kg to assist in the prevention of exertional myopathy [16].

All females over 5 kg were injected in the interscapular subcutis with a single 10 mg deslorelin implant (Suprelorin) (Table 2). The following procedures were undertaken on the females:

- checked for pouch young (PY) and if found, the joeys were then sexed and weighed,
- patch of hair shaved and cleaned above the shoulders at the site for the hormonal implant,
- numbered red ear tag placed in the right ear and a red arm band on the right arm.

The following procedures were undertaken on all the males:

- intravenous injection of Alfaxan^®^ to provide a deeper and more stable level of anaesthesia during surgery. Alfaxan^®^ was also useful because of its shorter period of action than Zoletil^®^ particularly when given intravenously,
- scrotum was shaved, cleaned and prepared for surgery,
- castrated males were also given an injection of lignocaine directly into the testes as a local anaesthetic,
- one numbered red ear tag was placed in their left ear,
- one numbered blue ear tag was placed in the right ear and blue arm band was placed on the right arm if the animal was being castrated,
- alternatively, one numbered green ear tag was placed in the right ear and a blue arm band on the left arm if the animal was being vasectomised,
- injection of engemycin, benacillin antibiotics to help prevent post-operative infection and an injection of metacam for additional for pain relief and a consequently less stressful recovery.

**Table 2:** Summary of census and veterinary procedure data.

**Demographic**	**Procedure **	**Number of Kangaroos**	
		2003 Program	2007 Program
All kangaroos (incl. pouch young)	Estimated number present at time of management intervention ^1^	194	286
All kangaroos	Kangaroos darted	124	187
Females > 5 kg	Deslorelin implant	80	107
Males > 50 kg	Vasectomy	0	19
Males < 50 kg	Vasectomy	0	7
Males > 50 kg	Orchidectomy	0	5
Males > 5 and < 50 kg	Orchidectomy	0	52

^1^ Includes pouch young that were captured but not treated.

#### 2.5.2. Processing

Male kangaroos over 5 kg bodyweight were surgically sterilised. Most male kangaroos over 50 kg bodyweight were vasectomised to avoid hormonal changes in dominant males, and most males under 50 kg bodyweight were castrated. In addition, for the purposes of comparison, seven sub-adult males <50 kg were vasectomised and five adult males >50 kg were castrated to determine whether any changes in behaviour could be observed which might be attributed to the sterilisation method for the two classes (sub adult and adult) over the period of this study.

Surgical cases or animals with significant dart wounds were injected with an intramuscular dose of Benacillin (Troy Laboratories, Smithfield, NSW, Australia) at a rate of 1 mL per 10 kg of bodyweight and an intramuscular dose of Engemycin (Intervet Australia, Bendigo, Victoria, Australia) at a dose rate of 10 mg/kg of the active component (oxytetracyline 100 mg/mL). Age, sex, weight, ear tag numbers, anaesthetic details, clinical assessment and procedures were recorded for each animal. When the surgery was completed, each animal was carried to the recovery area and allowed to revive from the anaesthetic. At this stage, the data sheet was given to the veterinary nurse at the shaded recovery area to complete as the animal moved into sternal recumbency and finally left the recovery area of its own accord. During the recovery phase, the animal was spray painted carefully on its back with non-toxic paint, colour coded to indicate the processing session. This was done to further identify which kangaroo had already been sterilised or implanted, to facilitate their behavioural data collection and prevent them from being recaptured during the next day or session.

### 2.6. Behavioural Observations

Behavioural observations were conducted for 6 weeks prior to the start of the 2007 program, for 8 weeks spanning the capture sessions, and for 6 weeks afterwards. Observations were made on two days each week with the aid of binoculars from a golf cart and occurred over 1 hour sessions: once at sunrise (0700–0800), and once just before sunset (1600–1700). 

Preliminary observations identified the relevant behaviours to be recorded, using social and agonistic behaviours previously described [17,18]. Sexual behaviours observed in males were ‘following and sniffing females’ and penis erection, and agonistic behaviours were fighting with other males, chest beating (male kangaroos beating chest repeatedly with fore paws), high walking and grunting noises. These were recorded each time they were performed by either the focal kangaroo or kangaroos within a group, and had to occur for at least 5 seconds to be recorded. Two bouts were recorded if there was a break of 15 or more seconds between behaviours. 

Two observational techniques were used to detect behaviours:

(a) Focal animal sampling: one adult and one sub-adult male kangaroo were chosen at random during each observation session. While it would have been preferable to observe the same target kangaroos over the whole period of the study, this was not possible due to the difficulty in locating and identifying particular individuals across the 100 hectare site.
(b) Continuous sampling: the mean frequencies of agonistic and sexual behaviours for all visible sub-adult and adult male and all mature female kangaroos in a mob were recorded. Mobs of at least 15 kangaroos were chosen for behavioural observations to increase the detection of interactive behaviours.

Observations of the kangaroos’ reactions to darting were recorded during capture sessions for administration of reproductive control measures. A “darting reaction score” of 1–4 was recorded for each kangaroo darting event (1: kangaroo showed a minimal reaction to the dart with no flight response; 2: kangaroo jumped at the time of impact, and/or showed ongoing irritation or annoyance to the presence of the dart; 3: the kangaroo demonstrated an immediate flight response; 4: the kangaroo demonstrated rapid or immediate collapse or bone fracture).

### 2.7. Statistical Analysis of Behavioural Observations

The rates of sexual and agonistic behaviour before, during and after the program were compared in both the focal and continuous sampling groups using a chi-squared analysis, including a Bonferroni adjustment for multiple comparisons. The ‘following and sniffing females’ sexual behaviour observed in adult males during the continuous sampling was analysed using a Kruskal-Wallis test. Both tests were analysed with the Minitab™ statistical analysis program.

## 3. Results

### 3.1. Reproduction and Demographic Effects

**Figure 2:**
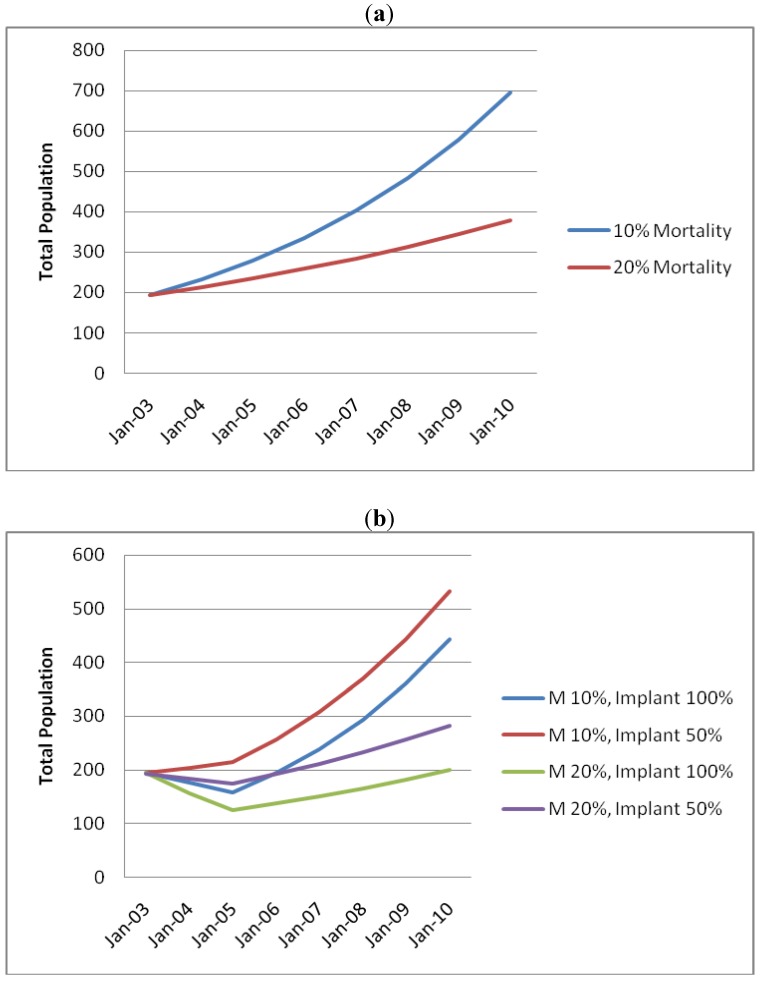
(**a**) Predicted kangaroo population growth with no intervention; (**b**) Predicted kangaroo population growth with intervention. M is annual mortality rate.

In early 2003, prior to any active population management, there were approximately 130 kangaroos present on the golf course, not including pouch young. Figure 2(a) shows estimates of projected population growth based on a birth rate of 0.3 of total population with two different mortality rates. This model assumes resources are not limiting and there is no immigration or emigration. Recruitment (birth rate) of 0.3 was based on the number of pouch young present as a proportion of the total kangaroo population at the time of the first management program and was based on an approximate pouch life of joeys of 12 months. 

Figure 2(b) shows predicted population growth with annual mortality rates of 10% and 20% for different implant responses; assuming the implant was effective in either all or 50% of animals for 12 months. 

In 2003, during the first control program there were approximately 194 kangaroos present, including pouch young, of which 181 (93%) were captured (Table 3). In November 2005, 26 months after the completion of the 2003 program, the estimated kangaroo population including pouch young was 174, including 37 pouch young, suggesting that the population growth was effectively zero over that period. This data is consistent with either of the two middle curves in Figure 2(b): 20% mortality and 50% implant efficacy, or 10% mortality with 100% implant efficacy. At the time of the second program in mid-2007, the population had grown to 286, of which 79 were pouch young (Table 4). This data is most consistent with the curve in Figure 2 representing 10% mortality with 100% implant efficacy.

**Table 3:** Demographic and management data for the 2003 program.

	**Above 5 kg**		**Pouch young**	**TOTAL**
	Males	Females	Unspecified	
Estimated animals present	47	87	60	**194 (100%)**
Total captured	44	80	57	**181 (93%)**
Estimated not captured	3	7	3	**13 (7%)**
Females implanted with deslorelin	-	80	-	**80 (92% of all females)**
Females with pouch young	-	60	-	**60**

**Table 4:** Demographic and management data for 2007 management intervention.

	**Above 5 kg**		**Pouch Young**	**Total**
	Males	Females	Unspecified	
Estimated animal present	82	125	79	**286 (100%)**
Total captured	72	115	79	**266 (93%)**
Estimated not captured	10	10	0	**20 (7%)**
Male orchidectomy	53	-	-	**53**
Males vasectomy	19	-	-	**19**
Females implanted with deslorelin	-	107	-	**107**
Females with pouch young	-	79	-	**79**
Mortality	11		2	**13**
Number with 2003 tags	40			**40**

**Figure 3:**
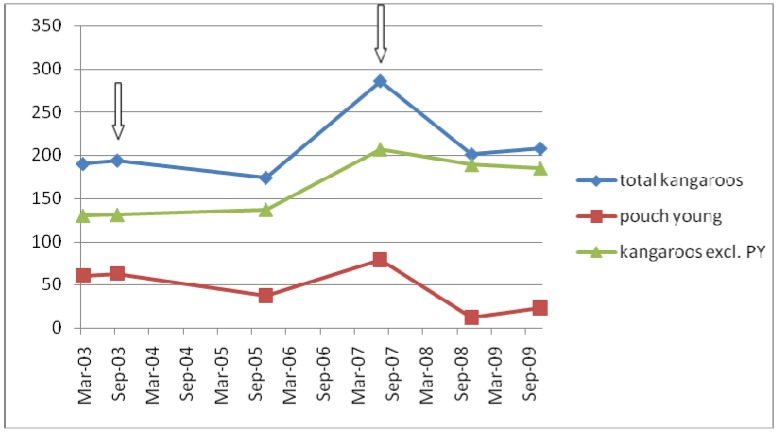
Graphical representation of changes in the total number of kangaroos, the number of pouch young and the number of kangaroos excluding pouch young (PY). The timing of management programs is indicated by arrows.

By late 2008, the population had declined to 201, with 12 pouch young and was only marginally greater in November 2009. The estimated number of pouch young decreased from 63 in 2003 to 37 in 2005 then increased to 79 in 2007, finally decreasing to 12 in November 2008. One year later, there were an estimated 23 pouch young, based on the 19 actually observed during the census.

These changes in total kangaroo population and observed pouch young are shown in Figure 3. 

The data is consistent with deslorelin implantation of female kangaroos causing a reduction in the average birth rate from 0.3, expressed as a proportion of total population, including pouch young, to close to zero by one year, and a return to 0.2 by 18 months. In comparison, the deslorelin implantation of females plus surgical sterilisation of males caused a more prolonged reduction in birth rate from 0.3 to 0.06 at 16 months and 0.1 at 28 months. The data is confounded to an extent by some immigration to, and emigration from the golf course of up to 20% of the population per year, based on observation of tagged *versus* untagged animals at the censuses [19]). 

### 3.2. Behaviour

#### 3.2.1. Agonistic Behaviours in Males

Agonistic behaviours tended to decrease post-treatment in castrated sub-adult males (<50 kg males) and were not seen at all in the vasectomised sub-adult males (P = 0.001) (Table 5a). Grunting was observed in untreated but not vasectomised or castrated sub-adult males. Grunting behaviour tended to decrease in castrated adult males compared to untreated and vasectomised adult males (P = 0.06). Fighting behaviour was reduced by vasectomisation and castration in sub-adults but not adults (Table 5b). 

Table 5(**a**) Agonistic behaviours in sub-adult and adult male kangaroos during focal and scanning animal sampling observations (analysed by Chi square test); (**b**) Occurrence of fighting behaviour seen in sub-adult and adult male kangaroos during continuous sampling observation sessions; (**c**) Sexually-related behaviours in sub-adult and adult male kangaroos during focal animal sampling observation sessions (analysed by Chi square test).

**Table:** (**a**) Agonistic behaviour

Age Group	Behaviour		Treatment			P Value		
			U*	V*	O*	U* *vs.* O*	U* *vs.* V*	O* *vs.* V*
Sub-adults	Fighting	Present	10	0	3	0.08	0.001	0.20
		Absent	18	29	25			
	Grunting	Present	8	0	0	0.007	0.006	N/A
		Absent	20	29	28			
Adults	Fighting	Present	5	8	2	0.68	0.76	0.21
		Absent	23	21	22			
	High walking	Present	5	4	1	0.33	0.97	0.55
		Absent	23	25	23			
	Chest beating	Present	3	6	1	0.76	0.66	0.21
		Absent	25	23	23			
	Grunting	Present	8	7	1	0.06	0.97	0.12
		Absent	20	22	23			

U*: Untreated, V*: Vasectomy, O*: Orchidectomy

**Table:** (**b**) Fighting behaviour

Age Group		Treatment			P Value		
		U*	V*	O*	U* *vs.* O*	U* *vs.* V*	O* *vs.* V*
Sub-adults	Present	6	0	0	0.01	0.01	0.99
	Absent	4	10	10			
Adults	Present	4	2	1	0.32	0.69	0,90
	Absent	6	8	9			

U*: Untreated, V*: Vasectomy, O*: Orchidectomy

**Table:** (**c**) Sexually-related behaviour

Age Group	Behaviour		Treatment			P Value		
			U*	V*	O*	U* *vs.* O*	U* *vs.* V*	O* *vs.* V*
Sub-adults	Following & sniffing	Present	8	0	1	0.03	0.006	0.664
		Absent	20	29	27			
	Erection	Present	10	0	1	0.007	0.001	0.68
		Absent	18	28	27			
Adults	Following & sniffing	Present	26	21	6	<0.001	0.122	0.002
		Absent	2	8	18			
	Erection	Present	18	19	4	0.002	0.99	0.001
		Absent	10	10	20			

U*: Untreated, V*: Vasectomy, O*: Orchidectomy

#### 3.2.2. Sexual Behaviours in Males

Both castrated and vasectomised sub-adult males had a reduced ‘following and sniffing females’ behaviour and had fewer erections of the penis compared to the untreated sub-adult males, but in the adult males this was only evident for the castrated and not the vasectomised animals (Table 5c). There was no ‘following and sniffing females’ behaviour observed in castrated adult male kangaroos, whereas there were 4.5 and 4.0 occurrences observed per hour for the vasectomised and untreated males, respectively (P < 0.001, Kruskal-Wallis test) during the continuous sampling. 

#### 3.2.3. Female Behaviour

Females that had been implanted spent longer in locomotory behaviour than those that had not (implanted 1.75 *vs.* unimplanted 1.35 log_n_ bouts/hour, SED 0.06, P = 0.04) and less time scanning (implanted 1.70 *vs.* unimplanted 2.85 log_n_ bouts/hour, SED 0.03, P < 0.001) and had fewer grooming bouts (implanted 2 *vs.* unimplanted 5 median bouts/hour, P < 0.001). There were no significant (P < 0.05) differences in feeding, standing or lying behaviour. 

#### 3.2.4. Animal Welfare Impact of Management Interventions

##### 3.2.4.1. Response to Darting

**Figure 4:**
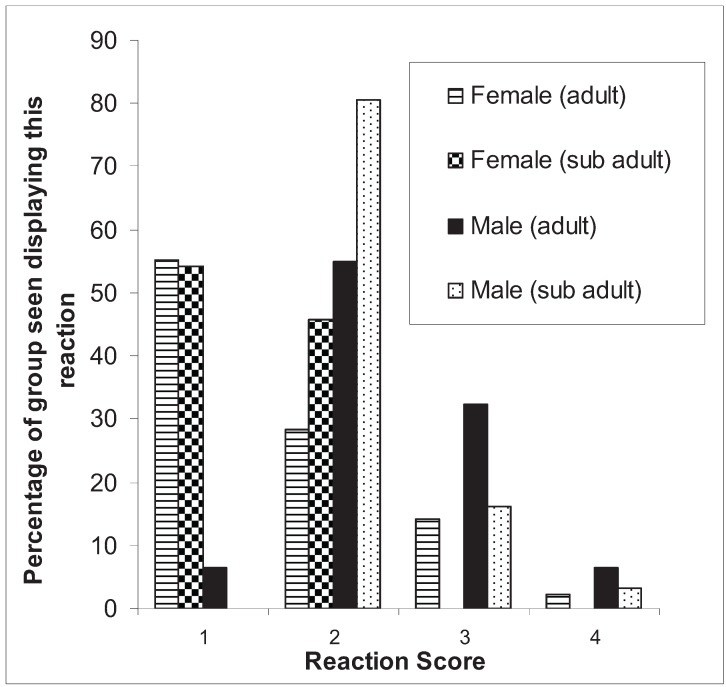
The kangaroos’ immediate physical reactions to being darted during the reproductive management interventions, 2007. 1: kangaroo showed a minimal reaction to the dart with no flight response; 2: kangaroo jumped at the time of impact, and/or showed ongoing irritation or annoyance to the presence of the dart; 3: the kangaroo demonstrated an immediate flight response; 4: the kangaroo demonstrated rapid or immediate collapse or bone fracture.

Individual responses of kangaroos to darting were recorded only in 2007. Of 187 kangaroos darted, 152 (81%) were given a “darting reaction score”. Most females received a score 1 or 2 and most males received a score 2 or 3 (Figure 4). Of the five kangaroos with the maximum darting reaction score of 4, four kangaroos (3%) collapsed immediately, presumably due to deposition of the anaesthetic agent directly into a vein or bone marrow, and one kangaroo suffered a fracture to the tibia, requiring euthanasia.

##### 3.2.4.2. Mortality

During both programs, there were darting or anaesthetic mortalities or incidents that required euthanasia. A summary of capture-associated mortalities is presented in Table 6. The animal capture rate (darted animals plus pouch young captured) for both projects was the same (93% of total estimated population), whereas the total mortality rate (as a % of total estimated population) during the 2007 project was less than one half (4.5%) that of the 2003 project (9.8%). Expressed as a percentage of animals captured, the respective mortality rates for 2003 and 2007 were 10.5% and 4.9%. 

Five deaths that occurred during recovery from anaesthesia in 2003 were attributed to hyperthermia/myopathy primarily due to high ambient temperatures (26°C or greater). Four animals were found dead up to 2 days after processing and the cause of death was investigated but unclear. Dart-related injuries included fracture of the femur, one spinal injury that required euthanasia and one pouch young that was struck by a dart and died. 

In the 2007 management intervention, two kangaroos died as a result of injuries caused by collision with trees during anaesthetic induction after darting. There were three drowning deaths or near-drowning incidents requiring euthanasia that occurred from anaesthesia when kangaroos were ambulatory, but still ataxic. 

**Table 6:** Summary of capture-associated mortalities from the 2003 and 2007 reproductive management plans.

Cause of death/reason for euthanasia	2003 (19 mortalities)		2007 (13 mortalities)		% of all deaths
	Died	Euthanased	Died	Euthanased	
Dart-related injury/fracture	0	5	0	1	19%
Trauma occurring during anaesthetic induction	0	0	0	2	6%
Hyperthermia/myopathy	5	0	1	0	19%
Death after anaesthetic recovery (unspecified cause)	4	0	3	0	22%
Anaesthetic death	0	0	1	0	3%
Drowning/near drowning	0	0	1	2	9%
Predation	2	0	0	0	6%
Untreatable disease	0	1	0	0	3%
Joey death (unviable orphan)	2	0	1	1	13%
Totals	13	6	7	6	100%

##### 3.2.4.3. Non-Lethal Injuries

Non-lethal injuries were generally limited to mild bleeding or haematoma formation at the dart wound requiring minimal veterinary treatment. Virtually all kangaroos experienced a prolonged recovery from anaesthesia (up to 4 hours), during which time they demonstrated significant ataxia and were observed to stand and collapse multiple times, before regaining full coordination and strength. 

Spearman’s rank correlation indicated a significant positive correlation between the dose rate and the time taken for a full recovery and release from the recovery area back onto the golf course. On average, mature females and males took 154 and 167 minutes, respectively, and sub-adult females and males took 182 and 177 minutes, respectively, to show a full recovery.

In both the 2003 and 2007 management programs, one pouch joey was inadvertently struck by a dart, became anaesthetised and was ejected from the mother’s pouch. In 2007, two pouch joeys were abandoned by their mothers during recovery from anaesthesia. All of these joeys were transferred to wildlife carers for hand-rearing and were successfully rehabilitated. 

##### 3.2.4.4. Financial Costs and Resource Effort

Each program required 15–20 personnel, including veterinarians, veterinary nurses and wildlife carers. As described in the Method, personnel were divided into three teams: the darting team, consisting of one veterinarian, one veterinary nurse, and one field biologist; the kangaroo transport teams consisting of three groups of two wildlife carers; and the veterinary procedures team, consisting of one veterinarian, two veterinary nurses and three wildlife carers. Additional volunteers (wildlife carers) assisted with animal handling and general duties. The total cost of the 2003 program was AU$18,900, and the 2007 program was AU$49,009. 

## 4. Discussion

### 4.1. Efficacy of Population Management Program

Based on our population censuses after each program, the reproductive control measures resulted in acceptable reduction of population fecundity, and therefore population growth. Although the census of November 2005 indicated a similar number of adults and sub-adult kangaroos present as were present prior to the 2003 management intervention, the number of pouch young was approximately one half. This result was consistent with our prediction of population number, based upon 100% efficacy of implants and approximately 15% natural attrition (deaths or emigration). At the time of the second management intervention, in mid-2007, the population had increased markedly, with an estimated 207 kangaroos (adults and sub-adults) present, plus approximately 79 pouch young. This is consistent with the expected return of normal fecundity following the loss of effect of the deslorelin implants, as estimated by Herbert and co-workers [5] to be approximately 18 months. However, a significant confounding factor was the potential for movement of kangaroos to and from the site due to numerous breaches in the perimeter fence. At the time of the 2003 management intervention and up to the November 2005 census, there was still a significant population of kangaroos inhabiting or using remnant grassland outside of the golf course, and ample anecdotal evidence of frequent use of breaches in the fence by kangaroos by the course staff and residents. Only 40 of 187 kangaroos darted during the 2007 management intervention had ear tags present from the 2003 management intervention, indicating that a significant proportion of the mature kangaroos present in 2007 were not processed in 2003, and therefore may have immigrated to the site since. In addition, a significant number (estimated to be 20 or more) of kangaroos with ear tags had been killed by motor vehicle strike on roads adjacent to the golf course over the intervening years. By comparison, only 18 of 95 adult kangaroos counted in the November 2008 census were untagged. Given that approximately 20 kangaroos were not captured in the mid-2007 management intervention, the figures are consistent with minimal immigration to the site over that time. Furthermore, urban development had almost entirely destroyed any remnant habitat for kangaroos outside of the golf course by mid-2007.

Our data suggest that deslorelin treatment of female kangaroos alone is sufficient to offer a temporary solution to population growth in eastern grey kangaroos. However, repeat treatments may be required every two years or so, depending upon rates of natural attrition. The addition of surgical (and therefore permanent) sterilisation procedures in male kangaroos was intended to increase the duration of reproductive suppression within the population beyond that provided by deslorelin implantation (of females) alone. Return of fecundity would then rely on the sexual maturation of untreated males (pouch young at the time of the 2007 management intervention), as well as loss of effect of implants in females. In a closed population, this combination is expected to result in a significant drop in the total kangaroo population, followed by a slow increase as normal fecundity returns. However, it is likely that the presence of even a few intact sexually mature males will obviate the effect of permanent sterilisation of other males, as females return to normal reproductive function. The use of levonorgestrel implants in female kangaroos is reported to give a longer period of reproductive suppression compared with deslorelin [6]. Currently, these implants are significantly more expensive than deslorelin implants (AU$250 *vs.* 60/implant), a factor which will influence cost/% reduction in fecundity/annum. Surgical sterilisation of females, such as by tubal ligation/transection or ovario-hysterectomy, is more invasive than in males and was not considered for financial, ethical and logistic reasons in this program. However, it does offer permanent reproductive control, and is worthy of consideration if financial considerations and other disadvantages are able to be addressed or mitigated. The current programs cost approximately AU$500/year/% reduction in birthrate for GnRH program alone and AU$1050/year/% reduction in birth rate for the deslorelin plus surgery program.

### 4.2. Animal Welfare Considerations

Anaesthesia and darting of wild animals invariably carry some risk [17]. These risks are associated with:

(1) Injury caused by the dart
(2) Injury or misadventure that occurs during induction or recovery from anaesthesia
(3) Anaesthesia and other veterinary procedures
(4) Predation as a result of impairment of appropriate flight or defence responses [18]

In our program, the mortality rate associated with dart injuries was significantly lower in the second management intervention (8% of all deaths in 2007) than in the first (25% of all deaths in 2003). With respect to the total number of animals actually darted, the figures are 0.5% (2007) and 4% (2003). This difference we attribute primarily to the greater accuracy and finer pressure adjustments of the Dan-Inject dart gun compared with the Montech Taipan 2000. In this program, the target kangaroos were used to human presence, their flight distances were short, and therefore darting distances were significantly shorter than could be expected of kangaroos that were not familiar with humans. This factor contributed significantly to the safety and efficiency of darting capture. We suspected an upward drift in flight distance over time. This could be due to the animals that were easier to dart being targeted first. Alternatively, it could arise from a learnt response to the stress of the darting events. Regular excursions to the site with simulated dart firing prior to a darting event would be one possibility to potentially reduce fear responses.

**Figure 5:**
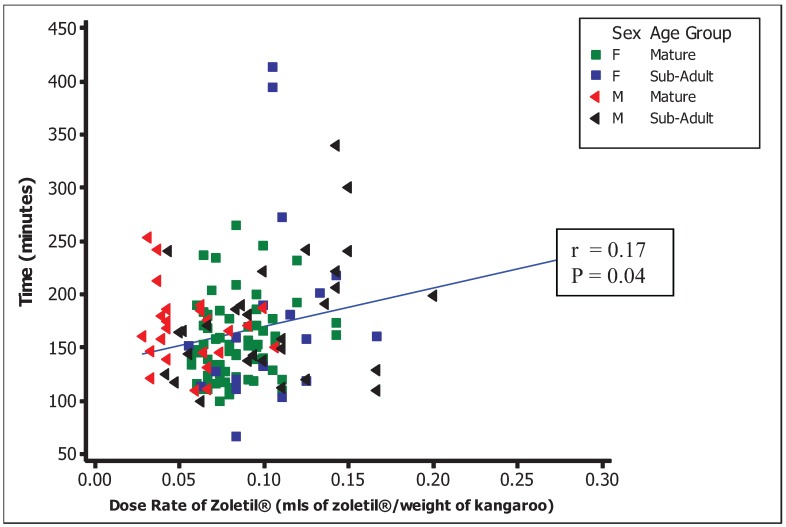
Time taken to full recovery* from anaesthetic (Zoletil^®^) during the 2007 intervention at the Pines golf course.

Injury and mortality associated with anaesthetic induction and recovery were significant. Rapid induction of anaesthesia with minimal excitement is desirable as it reduces the risk of injury and misadventure during the period between darting and recumbency and reduces the likelihood of an animal becoming lost when, or if, it takes flight in response to darting. An accurate weight estimate is essential to calibrate the dose. Tiletamine/zolazepam (Zoletil^®^, Virbac (Australia), NSW) is a preferred anaesthetic agent for darting of kangaroos because of its short average induction time [17,20,21]. However, it has some significant disadvantages, mainly associated with its prolonged and sometimes violent recovery phase. In addition, in our experience, the risk of serious or fatal hyperthermia in kangaroos anaesthetised with Zoletil is significant at ambient temperatures above approximately 24 °C, because of factors such as tachypnoea and exertion or muscular activity during induction and recovery. We therefore recommend that darting of wild kangaroos not be performed when ambient temperatures are expected to exceed 24 °C at any time between anaesthetic induction and complete recovery. Another factor contributing to poor animal welfare outcomes, and sometimes death of kangaroos, was physical injury that occurred during anaesthetic induction and recovery. Those occurring during anaesthetic recovery may be minimised by confining recovering kangaroos to hessian or shade cloth pens supported at each corner by posts protected by polystyrene padding. These provided some support if the kangaroo knocked into the sides during recovery, whilst minimising injuries. We also avoided excessive disturbance or noise during this phase and used removable eye masks to decrease the amount of light when kangaroos were recovering. Avoiding injuries during anaesthetic induction is somewhat more difficult, but may be minimised by a quiet and calm approach to darting, and avoiding disturbance of chasing darted kangaroos during the induction phase of anaesthesia. Various anaesthetic combinations, including the use of the reversible agents medetomidine and xylazine, were tried in our program, but resulted in unacceptably longer and less predictable anaesthetic induction times when used at recommended dose rates [16]. Consequently, we used Zoletil as our primary agent, despite the issues associated with recovery from anaesthetic [22,23]. In our study, recovery time was longer following higher anaesthetic dose rates (Figure 5). Further development of safe and rapid-induction anaesthetic regimes with superior recovery characteristics to Zoletil may significantly reduce morbidity and mortality associated with anaesthetic recovery. Other important considerationsrelatedto high-volume macropod anaesthesia include human safety issues, costs (of anaesthetic drugs and reversal agents) and the limitations of dart volume.

* Kangaroo was able to maintain balance and move away from the recovery area of its own accord.

#### 4.2.1. Animal Reaction to Darting

Most kangaroos displayed only a minimal reaction to the impact of the dart, suggesting that darting generally did not inflict significant pain. As this program involved a population of kangaroos highly habituated to close human presence, it is possible that different reactions would be observed if kangaroos were not as habituated to humans or vehicles. The most serious welfare impacts associated with darting and anaesthesia were those associated with recovery from anaesthesia, during which time kangaroos were prone to injury and hyperthermia, which in some instances led to death. 

#### 4.2.2. Short Term Behavioural Responses of Kangaroos to Reproductive Procedures

The results demonstrate that some agonistic behaviours, such as fighting and grunting, still occurred in the sub-adult male kangaroos given the orchidectomy procedure 6–8 weeks post surgery. However, compared with the males given the vasectomy procedure and with observations of untreated sub-adult males during the pre-treatment period, the frequency of these behaviours was reduced. 

Surgically-sterilised adult male kangaroos did not show a significant difference in agonistic behaviours during the post-treatment period of observation, neither was there any difference between these groups when comparing pre- and post-treatment observations. This may be due to the relatively short time between surgical procedures and the last set of observations, and persistence of significant levels of testosterone in castrated males. This finding is similar to the results of a study of rock hyrax [24], in which adult males given the orchidectomy procedure still displayed agonistic behaviours when approached by other males, but they were never seen to initiate them. Such behavioural inertia is to be expected given the significant time and learning required for the development of the behaviour. We expect that longer-term observations of our population of kangaroos may show a decrease in initiated agonistic behaviours in castrated male kangaroos, although our current (short-term) data do not demonstrate this.

In contrast to our observations, other studies [25,26] suggest that the display of sexual behaviours is infrequent in sub-adult male eastern grey kangaroos and that only dominant males will ‘follow and sniff’ the females. We observed a decrease in the sexual behaviours of surgically sterilised sub-adult males in the post-treatment period, when compared with the control (pre-treatment) period. The reason is unclear, but it may be a function of the relatively short period of observation post-treatment or low sampling effort. In the adult males given the orchidectomy procedure, sexual behaviours decreased significantly during the post-treatment observation period, and were almost completely absent by the end of the observation period. The plasma levels of testosterone in the male eastern grey kangaroos following this procedure have not been reported in the literature, although it decreases after just 5 days in tammar wallabies (*M*. *Eugenii*) [27].

Staker [28] found that macropodid males post-vasectomy did not have decreased agonistic behaviours, which is consistent with the fact that vasectomy does not significantly alter the level of circulating androgens in the body. This is consistent with our observations and those of Bolitho *et al.* [29], who reported similar results in male eastern grey kangaroos given a vasectomy. 

In the females, the longer time spent walking may indicate a less cohesive social group, reflecting reduced sexual activity in both males and females. The need for an effective perimeter fence is evident, particularly if there is a resident population outside the target population, as in this study. In the females there was also less time spent scanning, possibly for potential mates, and less time self-grooming, perhaps reflecting less tension in the group as a result of reduced sexual activity. These changes were not observed in previous research using deslorelin in kangaroos [30].

Our study targeted the entire population on the golf course, and approximately 90% of animals were actually treated. This appeared effective in controlling population for several years. If a smaller proportion had been treated, or there had been greater incursion into the course for example as a result of a drought, the duration of reproductive control would diminish. 

## 5. Conclusions

Population management plans involving non-lethal reproductive manipulation are increasingly required to manage landlocked wildlife populations. The development and assessment of effective and humane management techniques are essential if wildlife managers are to appropriately respond to community expectations for compassionate and ethical solutions. Our study demonstrates that although medium-scale kangaroo reproductive management programs are, or can be, effective and achievable, there are nevertheless significant risks to animal welfare that must be mitigated if such programs are to be utilised widely. Key issues affecting animal welfare are generally associated with induction of, and recovery from anaesthesia, during which time kangaroos are susceptible to misadventure and injury. These risks can be mitigated to some degree by minimisation of disturbance and rapid induction of anaesthesia, appropriate confinement during recovery, use of well-trained and experienced personnel, and avoiding darting during warmer months. In spite of the best preparation and precautions we suggest that mortality rates in kangaroos of 5–10% may be expected until better anaesthetic regimes for field darting and chemical restraint become available. 
